# Evaluation of the MAGLUMI HIV Ab/Ag combi test for the detection of HIV infection

**DOI:** 10.1186/s12985-024-02565-x

**Published:** 2024-11-13

**Authors:** Chunling Wang, Jie Rao, Zhonggang Fang, Hongwei Zhang, Jun Yin, Tinghua Li, Chen Zhang

**Affiliations:** 1 Center of Clinical Laboratory Medicine, Zhongda Hospital, Southeast University, Nanjing, 210009 People’s Republic of China; 2 Research & Development Department, Shenzhen New Industries Biomedical Engineering Co., Ltd. (Snibe), No.23, Jinxiu East Road, Pingshan District, Shenzhen, 518122 People’s Republic of China

**Keywords:** HIV, Diagnosis, Chemiluminescence immunoassay, MAGLUMI, Seroconversion

## Abstract

**Background:**

Human immunodeficiency virus (HIV) infection screening and diagnosis are critical to control the HIV epidemic. Testing for anti-HIV antibodies (Ab) and antigens (Ag) in blood samples is the first step to screen people who have been potentially exposed to the virus. This study aimed to evaluate the performance of the MAGLUMI HIV Ab/Ag Combi for detection of HIV antibodies and antigens.

**Methods:**

We used residual samples to assess the diagnostic specificity and sensitivity of the MAGLUMI HIV Ab/Ag Combi retrospectively. All samples that met the test criteria were tested with the MAGLUMI HIV Ab/Ag Combi according to manufacturer’s instruction. Results of the MAGLUMI HIV Ab/Ag Combi were compared with the Architect HIV Ag/Ab Combo test.

**Results:**

The specificity of the MAGLUMI HIV Ab/Ag Combi was 99.85% in 5,057 unselected blood donors and 100.00% in 213 hospitalized patient samples, respectively. The sensitivity of the Test in 614 HIV-1 Ab, HIV-1 Ag or HIV-2 Ab positive samples was 100.00%. Seroconversion sensitivity from results of 30 panels was comparable between the MAGLUMI HIV Ab/Ag Combi and the Architect assay.

**Conclusions:**

The reactivity of the MAGLUMI HIV Ab/Ag Combi test is comparable to the Architect HIV Ag/Ab Combo assay.

**Supplementary Information:**

The online version contains supplementary material available at 10.1186/s12985-024-02565-x.

## Introduction

In 2023, about 1.3 million new HIV infections occurring and approximately 630 thousand people died from HIV-related causes [1, 2]. Even though there is no cure or effective vaccine valid for HIV infection [3], antiretroviral therapy (ART) is able to turn HIV infection into a chronic disease, provide longer lives for patients and reduce HIV transmission [4]. The challenge is that HIV-infected individuals are unaware of their status until the later stages and fail to receive timely treatment [5]. This is due to the latency of HIV infection that in the first few weeks after being infected people may have no symptoms or only have an influenza-like illness, following with a chronic phase persisting no symptoms or mild ones for years [5]. Without treatment, people with HIV infection can develop severe illnesses including tuberculosis, cryptococcal meningitis, pneumocystis pneumonia and cancers [6–9]. Therefore, the detection and management of early HIV infection is critical to improve patient health, reduce their additional medical burden and control the risk of onward viral transmission [10]. However, the diagnosis rate of HIV infections is still far from satisfactory, about 5.5 million people did not know they were living with HIV in 2022 [2].

The US Centers for Disease Control and Prevention (CDC) recommends that everyone aged between 13 and 64 get tested for HIV at least once as part of routine health care. People with certain risk factors should get tested at least once a year [11]. Sexually active Men who have sex with men (MSM) were recommended to get more frequent testing (every 3 to 6 months) [11]. Testing for both of antibodies (Ab) and antigens (Ag) in serum and plasma specimens is considered to be the first step to screen the people who have been potentially exposed to HIV [12, 13]. Additionally, a confirmatory HIV test and/or subsequent HIV RNA testing are required for specimens with a positive result or a suspected very early infection [12, 13]. Although the HIV RNA test is useful for a recent high-risk HIV exposure, confirming a result from another HIV test and monitoring viral load [14, 15], HIV RNA test is not recommended to initially screen HIV infection due to its expensive fees with only slightly shortened window period (Supplementary Figure S1) [16, 17].

The current commercial tests for HIV infection diagnosis widely used mainly belong to the Ab or Ag/Ab tests. Ag/Ab assays detect HIV antibodies together with the p24 antigen, allowing for more sensitive detection of recent HIV infection and reduction of the window period by comparing with Ab assays, which only detect antibodies [18]. In recent years, several new automated HIV Ag/Ab assays have been brought to market, including Roche Elecsys HIV Duo assay, Beckman Access HIV Ag/Ab combo, Beckman Access HIV combo V2 assay and Snibe MAGLUMI HIV Ab/Ag Combi (MAGLUMI) [19–21]. MAGLUMI is based on the double-antigen sandwich principle and uses recombinant HIV-1/HIV-2 antigens to detect HIV-1 and/or HIV-2 antibodies, in the meanwhile, anti-HIV-1 p24 monoclonal antibodies are used to detect HIV-1 antigens. In this study, we evaluated the clinical performance of the MAGLUMI to verify whether the test is suited for HIV infections.

## Materials and methods

### MAGLUMI HIV Ab/Ag combi test

MAGLUMI is a two-step sandwich chemiluminescence immunoassay. The first step: The sample, the magnetic microbeads and N-(4-Aminobutyl)-N-ethylisoluminol (ABEI) are mixed thoroughly and incubated. Here the magnetic microbeads coated with anti-HIV-1 p24 monoclonal antibodies and HIV-1/HIV-2 recombinant antigens (recombinant gp41 of HIV-1 and gp36 of HIV-2). The ABEI labeled with anti-HIV-1 p24 monoclonal antibodies. The antibodies to HIV-1 and/or HIV-2 present in the sample bind to the HIV-1 and HIV-2 recombinant antigens to form a complex, and the HIV-1 p24 antigens present in the sample bind to the anti-HIV-1 p24 monoclonal antibodies. The second step: After washing, ABEI labeled with HIV-1 and HIV-2 recombinant antigens (recombinant gp41 of HIV-1 and gp36 of HIV-2) are added, and bind to the complex. Following another wash cycle, the rest unbound materials are removed, the Starter 1 + 2 are added to initiate a flash chemiluminescent reaction. The light signal is measured by a photomultiplier as relative light units (RLUs), which is indicative of the concentration of HIV p24 antigens and antibodies against HIV-1 and/or HIV-2 present in the sample. Biotin is excluded in the assay design and there is no interference when biotin concentration is not exceeding 50 µg/mL. All samples in this study were tested with MAGLUMI according to manufacturer’s instruction.

### Blood donor and patient samples

A total of 5,057 blood donor samples (serum or plasma) with negative status for the Architect HIV Ag/Ab Combo (Architect) were collected from two blood donation centers in Germany (Table 1). These samples were directly used for assessing the performance of diagnostic reagents and the donation centers did not provide the HIV RNA results for this study, although we know that HIV-1 RNA test was mandatory in regular blood donation centers in Germany. All samples were tested with MAGLUMI and the samples with a result greater than or equal to 1.00 AU/mL (≥ 1.00 AU/mL) were considered to be positive and retests would be performed.

**Table 1:** Summary of samples included in performances analyses for the MAGLUMI HIV Ab/Ag Combi

Samples for specificity evaluation	5057 frozen blood donor samples
	213 frozen hospitalized patients samples
	110 frozen potentially cross reactive samples
	27 samples with potentially interfering substances
Samples for sensitivity evaluation	614 HIV known positive samples (459 HIV-1 Ab, 105 HIV-2 Ab, 50 HIV-1 p24 Ag)
	30 seroconversion panels
	NIBSC HIV-1 p24 antigen WHO international standard 90/636
	HIV Ab China national reference panel
	p24 antigen China national reference panel

*HIV*, human immunodeficiency virus; *Ab*, antibodies; *Ag*, antigens

Blood donor and HIV positive samples used in this study were all residual samples with known test values. A total of 614 HIV-positive samples were included in the sensitivity calculation, 459 HIV-1 Ab, 105 HIV-2 Ab and 50 HIV-1 Ag samples (Table 1). Samples were either initially tested with Architect, Abbott PRISM or BioMérieux VIDAS HIV-DUO. Initially reactive samples were confirmed for antibody presence by Bio-Rad Geenius HIV-1/2 Confirmatory Assay. The presence of HIV-1 antigen p24 for these samples were confirmed by DiaSorin LIAISON XL Murex HIV Ab/Ag, BIO-RAD BioPlex 2200 HIV Ag/Ab assay or Roche Elecsys HIV-1 Ag. Among the HIV-positive samples, 436 HIV-1 Ab, 55 HIV-2 Ab and all 50 HIV-1 Ag samples have test results of Architect. Furthermore, the HIV-positive status of all HIV-1 Ab and Ag positive samples were confirmed by HIV-1 RNA test on Abbott m2000 RealTime System. Among these HIV positive samples, 270 HIV-1 and 12 HIV-2 Ab positive samples were with known subtype respectively. Specifically, 10 HIV-1 subtypes and 13 HIV-1 CRFs were included in these HIV-1 positive samples with known subtype.

Samples from 213 consecutive hospitalized patients were used to evaluate the analytical specificity of the test (Table 1). All these patients have a clinical diagnosis of other diseases and no history of clinical symptoms and epidemiology related to HIV infection. Samples of these patients were selected and then tested by both MAGLUMI and Architect.

### Seroconversion sensitivity

30 commercially available HIV seroconversion panels representing a total of 356 samples were tested (Table 1). The panels were: SCP-HIV1-002, SCP-HCV1-007 (Biomex GmbH, Heidelberg, Germany), HIV 6244, HIV 6248, HIV9011, HIV9012, HIV9013, HIV9016, HIV9018, HIV9020, HIV9021, HIV9022, HIV9023, HIV9030, HIV9031, HIV9034, HIV9076, HIV9077, HIV9079, HIV9089, HIV9096, HIV12008 (ZeptoMetrix, Buffalo, NY, USA), PRB945, PRB955, PRB963, PRB966, PRB968, PRB969, PRB973, 0600–0271 (SeraCare Inc., Milford, MA, USA). The panels were evaluated between MAGLUMI and Architect to assess how early two assays could detect infection. Numbers of positive samples and the first positive day of each panel were determined and compared to evaluate the sensitivity on seroconversion. The shorter undetectable days (number of days between the first Ab/Ag positive day and the first blood day) or the more detectable panel samples mean the test detected HIV Ab/Ag earlier. Data of Architect were collected from the seroconversion panel datasheets.

### Endogenous sample interference

In total 110 samples with potentially cross reacting substance/ agents including samples generated from patients with auto-immune diseases, pregnant women, patients with hyper IgG/IgM, flu patients, dialysis patients and patients with infectious diseases were evaluated (Table 1). 27 samples with interfering substances including hemolytic, lipemic and icteric were assessed (Table 1).

### Analytical sensitivity in international standard and national reference

The twofold dilution series of the NIBSC HIV-1 p24 antigen WHO international standard (code: 90/636) were tested in parallel on MAGLUMI and Architect (Table 1). HIV-1 Ab (Lot: 370,045–201901) and p24 antigen (Lot: 220,015–201906) China national reference panels were also tested on MAGLUMI (Table 1).

## Statistical analysis

Calculation of proportions and corresponding Wilson 95% confidence intervals were performed using Analyse-IT v4.81 (Leeds, United Kingdom). Analysis of differences between various groups of MAGLUMI AU values and correlation analysis of the results obtained on HIV-1 Ab, HIV-2 Ab and HIV-1 Ag positive samples were performed by GraphPad Prism Software (San Diego, CA, USA). Differences between various groups of MAGLUMI AU values were determined using the Kruskal–Wallis test. Linear correlations of the results obtained on HIV-1 Ab, HIV-2 Ab and HIV-1 Ag positive samples between MAGLUMI and Architect were determined by the Pearson correlation coefficient.

## Results

### Diagnostic specificity and sensitivity

Among 5,270 Ag/Ab negative samples from 5,057 blood donors and 213 hospitalized patients, 8 gave presumed false positive results on MAGLUMI. This resulted in a diagnostic specificity of 99.85% (5,262/5,270) (Table 2). After centrifuged and transferred the clarified specimen to a second tube for retesting, seven samples were repeatedly below the MAGLUMI cutoff (Supplementary Table S1).

**Table 2:** Performance characteristics of the MAGLUMI HIV Ag/Ab Combi on samples from blood donors and HIV-negative hospitalized patients

Measure	Calculation	Estimate	95% CI
Specificity	5,262/5,270	99.85%	99.70%-99.92%
Sensitivity	614/614	100.00%	99.38%-100.00%
FPR	8/5,270	0.15%	0.08%-0.30%
FNR	0/614	0.00%	0.00%-0.62%
PPV	614/622	98.71%	97.48%-99.34%
NPV	5,262/5,262	100.00%	99.93%-100.00%
Accuracy	5,876/5,884	99.86%	99.73%-99.93%

*FPR*, false positive rate; *FNR*, false negative rate; *PPV*, positive predictive value; *NPV*, negative predictive value; *CI*, confidence interval

All 459 HIV-1 Ab and 105 HIV-2 Ab positive samples included 282 samples with a known HIV genotype as well as 50 HIV-1 Ag positive samples were found positive with MAGLUMI which presented a 100.00% diagnostic sensitivity (Table 3). Both MAGLUMI and Architect are chemiluminescent immunoassays and the relative light units correlate with the concentration of the measured substance in a certain range, which gives them the potential for determining recent infection based on different values [22]. So we did correlation analysis of the results with specific test values obtained by both assays on HIV-1 Ab, HIV-2 Ab and HIV-1 Ag positive samples respectively. We found a very poor correlation of HIV antibody values between MAGLUMI and Architect (Suppl. Figure S2). However, a good correlation of HIV-1 Ag values was observed (Suppl. Figure S2).

**Table 3:** Sensitivity of the MAGLUMI HIV Ab/Ag Combi in HIV-1 Ab, HIV-1 Ag and HIV-2 Ab positive samples

	Genotype	# samples	Positive	Negative
HIV-1 Ab positive	-	189	189	0
	A	13	13	0
	A1	11	11	0
	B	22	22	0
	C	3	3	0
	D	22	22	0
	CRF01_AE	21	21	0
	CRF02_AG	29	29	0
	CRF03	4	4	0
	CRF06	4	4	0
	CRF09	2	2	0
	CRF11	16	16	0
	CRF13	12	12	0
	CRF14	3	3	0
	CRF18	31	31	0
	CRF22	4	4	0
	CRF25	1	1	0
	CRF26	1	1	0
	CRF37	2	2	0
	F	8	8	0
	F2	15	15	0
	G	25	25	0
	H	4	4	0
	J	3	3	0
	K	7	7	0
	Group O	7	7	0
HIV-1 Ag positive	-	50	50	0
HIV-2 Ab positive	-	93	93	0
	A	9	9	0
	B	3	3	0
Total		614	614	0

*HIV*, human immunodeficiency virus; *Ab*, antibodies; *Ag*, antigens; *CRF*, circulating recombinant form

### Seroconversion sensitivity

In this study, 30 commercially available HIV seroconversion panels were tested to evaluate seroconversion sensitivity of MAGLUMI. For each panel, numbers of positive samples and delayed days of the first positive days based on serological test results were compared (Table 4, supplementary Table S2). Comparing with Architect, MAGLUMI detected one more positive sample in two panels and the same samples in the rest panels. Therefore, MAGLUMI detected slightly more panel samples (125 of 356) compared with Architect (123 of 356) on 30 seroconversion panels (Table 4). Total delayed days since the first blood draw of 30 panels tested were slightly fewer in MAGLUMI (1,105 days) compared with Architect (1,115 days), which indicated that MAGLUMI detected HIV infection as early as Architect (Supplementary Table S2). It is worth noting that one panel (SCP-HIV1-007) starts with a negative sample, seroconverts and turns back to negative, and the same effect was seen in both assays. 114 samples from 27 seroconversion panels were defined as early seroconversion HIV samples. Early seroconversion samples were defined in the Common Technical Specifications (2002/364/EC and amendments) as p24 antigen and/or HIV RNA positive, not recognized by all of the antibody screening tests, and indeterminate or negative confirmatory assays [23]. Among these early seroconversion HIV samples, 60 and 58 were detected as positive with MAGLUMI and Architect, respectively (Supplementary Table S3). In conclusion, based on numbers of positive samples and delayed days of the first positive days, MAGLUMI performed comparable on seroconversion sensitivity compared with Architect.

**Table 4:** Seroconversion sensitivity: comparison of MAGLUMI HIV Ab/Ag Combi and Architect HIV Ag/Ab Combo with 30 seroconversion panels

Panel ID	Panel samples	MAGLUMI HIV Ab/Ag Combi	Architect HIV Ag/Ab Combo
PRB945	6	3	3
PRB955	5	4	4
PRB963	7	2	2
PRB966	10	3	3
PRB968	10	4	4
PRB969	10	4	4
PRB973	4	2	2
HIV6244^*^	13	1	1
HIV6248	7	2	2
HIV9011	11	2	2
HIV9012	8	3	3
HIV9013	7	2	1
HIV9016	10	2	2
HIV9018	11	3	3
HIV9020	22	3	3
HIV9021	17	4	4
HIV9022^*^	8	1	1
HIV9023	22	3	3
HIV9030	16	3	3
HIV9031	19	4	3
HIV9034	13	3	3
HIV9076^*^	9	3	3
HIV9077^*^	24	13	13
HIV9079	25	17	17
HIV9089	6	3	3
HIV9096	6	5	5
HIV12008	13	5	5
SCP-HIV1-002	20	11	11
SCP-HIV1-007^**^	9	4	4
0600–0271	8	6	6
Total on 30 panels	356	125	123

^*^ HIV6244-14, -15, HIV9022-9, HIV9076-07, HIV-9077–23, -26, -27, -28 and -29 were exhausted and therefore not tested. For both tests the same panel samples were compared

^**^ SCP-HIV1-007 starts with a negative sample, seroconverts and turns back to negative

*HIV*, human immunodeficiency virus; *Ab*, antibodies; *Ag*, antigens

### Analytical specificity

All 137 samples with potentially cross reacting substances/agents or interfering substances were negative with MAGLUMI (Table 5). The analytical specificity on 137 samples with potential cross reacting/interfering substances was 100.00% (137/137).

**Table 5:** Analytical specificity of MAGLUMI HIV Ab/Ag Combi in samples with potentially cross reacting substances/agents and interfering substances

Cross reacting and interfering substances/agents	Positive	Negative	
Cross reacting substances/agents	Auto-immune diseases	0	20
	Pregnant women	0	7
	Hyper IgG/IgM	0	8
	Flu patients	0	7
	Patients with chronic kidney disease/dialysis insufficiency (dialysis patients)	0	7
	Anti-TP positive	0	7
	Anti-HEV positive	0	7
	Anti-HCV positive	0	7
	Anti-EBV positive	0	6
	Anti-CMV positive	0	7
	Anti-VZV positive	0	5
	Anti-HSV-1/2 positive	0	6
	Anti-HAV positive	0	6
	HBsAg positive	0	6
	Anti-HBc positive	0	4
Interfering substances	Hemolytic Low (0.6 g/dL)	0	3
	Hemolytic Medium (1.0 g/dL)	0	3
	Hemolytic High (1.9–2.0 g/dL)	0	3
	Lipemic Low (213–270 mg/dL)	0	3
	Lipemic Medium (440–556.3 mg/dL)	0	3
	Lipemic High (872.9–1083 mg/dL)	0	3
	Bilirubin (lcteric) Low (14.4–15.2 mg/dL)	0	3
	Bilirubin (lcteric) Medium (20.8 mg/dL)	0	3
	Bilirubin (lcteric) High (30.1–31.7 mg/dL)	0	3
Total		0	137
analytical specificity			100.00%
95% CI			97.27%-100.00%

*HIV*, human immunodeficiency virus; *TP*, *Treponema pallidum*; *HEV*, hepatitis E virus; *HCV*, hepatitis C virus; *EBV*, Epstein-Barr virus; *CMV*, cytomegalovirus; *VZV*, varicella-zoster virus; *HSV*, herpes simplex virus; hepatitis A virus; *HBsAg*, hepatitis B surface antigen; *HBc*, hepatitis B core antigen

### Analytical sensitivity

Both MAGLUMI and Architect detected the HIV-1 p24 antigen international standard with a similar sensitivity (Table 6). In this evaluation, the analytical sensitivity of both assays was 0.78 IU/mL which is in compliance with the common technical specification (CTS): ≤ 2 IU/mL. In addition, MAGLUMI detected both the HIV-1 Ab and p24 antigen China national reference panels with an acceptable analytical sensitivity (Supplementary Table S4). In p24 antigen national reference evaluation, the analytical sensitivity of the assay was 1.153 IU/mL which was in compliance with the CTS: ≤ 2.5 IU/mL. The obtained results of HIV-1 Ab (Limit of Detection) LoD and HIV-1 p24 antigen linearity as well as LoD were summarized in Supplementary Table S5.

**Table 6:** Comparison of analytical sensitivity in the NIBSC HIV-1 p24 antigen WHO International Standard 90/636 standard obtained with the MAGLUMI HIV Ab/Ag Combi and the Architect HIV Ag/Ab Combo. The mean value showed in the table were generated from three individual measurements

NIBSC 90/636 concentration IU/mL	MAGLUMI HIV Ab/Ag Combi AU/mL	Architect HIV Ag/Ab Combo S/Co
25.00	30.440	26.99
12.50	14.710	13.58
6.25	6.787	6.82
3.13	3.478	3.58
1.56	1.912	1.95
0.78	1.153	1.05
0.39	0.891	0.65
0.2	0.783	0.35
0.1	0.706	0.18
0.05	0.538	0.14

*HIV*, human immunodeficiency virus; *Ab*, antibodies; *Ag*, antigens; *IU*, international unit; *AU*, arbitrary unit; *S/CO*, signal/cut-off

## Discussion

This article reports the sensitivity and specificity of MAGLUMI for use on the MAGLUMI series fully-auto chemiluminescence immunoassay analyzer. The results showed that MAGLUMI had a specificity of 99.85% (5,262/5,270) in samples from blood donors and hospitalized patients, and a sensitivity of 100.00% (614/614) in HIV positive samples. The test also possessed a comparable seroconvertion sensitivity with Architect. In addition, we observed a high analytical specificity and sensitivity by evaluating the influence of factors including interfering substances, international standard, national reference, complement interference and serum to plasma equivalence. Based on the Common Technical Specifications 2002/364/EC and amendments, it can be concluded that MAGLUMI has a reasonably good specificity and sensitivity.

Among samples used to assess diagnostic specificity, eight gave presumed false positive results and one of them remained positive result after centrifugation. Actually, Fibrin were observed in all eight presumed false positive samples, emphasizing on the correlation between sample condition, necessary reparation for analysis and reliable results. One limitation of this study is that lack of information on HIV RNA status of blood donors, hospitalized patients and HIV-2 Ab samples. Therefore, the presumed false positive results detected among these samples have not been confirmed.

HIV is a highly variable virus characterised by extensive genetic heterogeneity [24, 25], which brings a challenge to the development of vaccines and pan-genotypic treatments [26], and also requires diagnostic tests applicable to the detection of all genotypes. HIV can be divided into 2 lineages, HIV-1 and HIV-2 [27]. HIV-1 is the predominant lineage in the global epidemic accounts for 95% of infections. Based on the differences in nucleic acid sequence of *env* gene, HIV-1 is divided into 4 groups (M, N, O, and P), among which the main group is M group including at least 9 subtypes (A-D, F–H, J, K) and various circulating recombinant forms (CRFs) [27–29]. HIV-1 M group infection is the most prevalent globally, subtypes like C, B, A and CRFs like CRF02_AG, CRF01_AE are widely distributed worldwide and account for a great proportion of the totality of HIV cases [24]. In this study, 270 HIV-1 Ab positive samples with 10 subtypes and 13 CRFs comprehensively covered most of the major subtypes. In addition, 12 HIV-2 Ab positive samples with subtype A or B were evaluated in this study. However, the detection rates of other HIV gene subtypes using this assay need to be verified.

MAGLUMI is an Ag/Ab chemiluminescence immunoassay (CLIA). The CLIA has been greatly developed in the past several decades and extensively used in diagnosis of infectious disease including HIV, hepatitis B and hepatitis C virus [30–32]. In some high-volume clinical laboratories, the CLIA is replacing the traditional EIA method in some high-volume clinical laboratories due to its technical simplicity, full automation and greater positive predictive value [33]. Recombinant protein gp41 of HIV-1, gp36 of HIV-2 and anti-HIV-1 p24 monoclonal antibody are widely used for the development with HIV Ag/Ab assays [19–21]. Besides, Beckman Access HIV combo V2 assay allows extra detection of HIV-2 p26 Ag [21]. It is noteworthy that a recent study showed the new Access HIV Ag/Ab combo assay exhibited better results of viral lysates and early detection on seroconversion panels compared with Architect and Roche Elecsys HIV Duo [20]. Therefore, to evaluate performance of MAGLUMI more comprehensively, it is necessary to compare its performance with more HIV Ag/Ab assays, especially the more sensitive assays approved in recent years.

Several studies claimed that very low S/CO values predict false-positive results and the false positive rate is reduced when S/CO increases [34–36]. In addition, current status of HIV infection could also be predicted by the signal-to-cutoff (S/CO) ratio derived from HIV Ag/Ab tests [22]. However, variable optimal S/CO values generated from different HIV Ag/Ab tests should be taken into account when predicting true HIV infection status. We observed a good correlation of positive Ag results, but poor correlations of both HIV-1 and HIV-2 Ab between MAGLUMI and Architect even though both tests performed high specificity and sensitivity. One possible reason could be differential material ratios the two tests used.

The Global Health Sector Strategies (GHSS) promote the disease-specific goal to end AIDS by 2030 [37]. To achieve this ambitious goal, by 2025, 95% of all people living with HIV should know their HIV status, 95% of all people with diagnosed HIV infection should be receiving sustained antiretroviral therapy and 95% of all people on treatment should have viral suppression by 2025 [2, 38]. However, only 86% of all people living with HIV knew their HIV status in 2022 [2]. “The first 95” target is fundamental to these ambitious goals, this way the infection can be timely monitored, controlled and treated to enable people living with HIV to avoid the further development of serious complications. MAGLUMI showed a comparable reactivity with Architect, which makes it have the potential to contribute to reaching “The first 95” target.

## Supplementary Information

Supplementary material 1.

Supplementary material 2.

Supplementary material 3.

Supplementary material 4.

Supplementary material 5.

Supplementary material 6.

Supplementary material 7.

## Data Availability

No datasets were generated or analysed during the current study.
