# Supplementary material for: Evaluation of the MAGLUMI HIV Ab/Ag combi test for the detection of HIV infection
Source: Virol J. 2024 Nov 13;21:290. doi: 10.1186/s12985-024-02565-x (PMC11562348; doi:10.1186/s12985-024-02565-x)
Supplement: Supplementary file 1 — Supplementary material 1. [file 12985_2024_2565_MOESM1_ESM.docx]

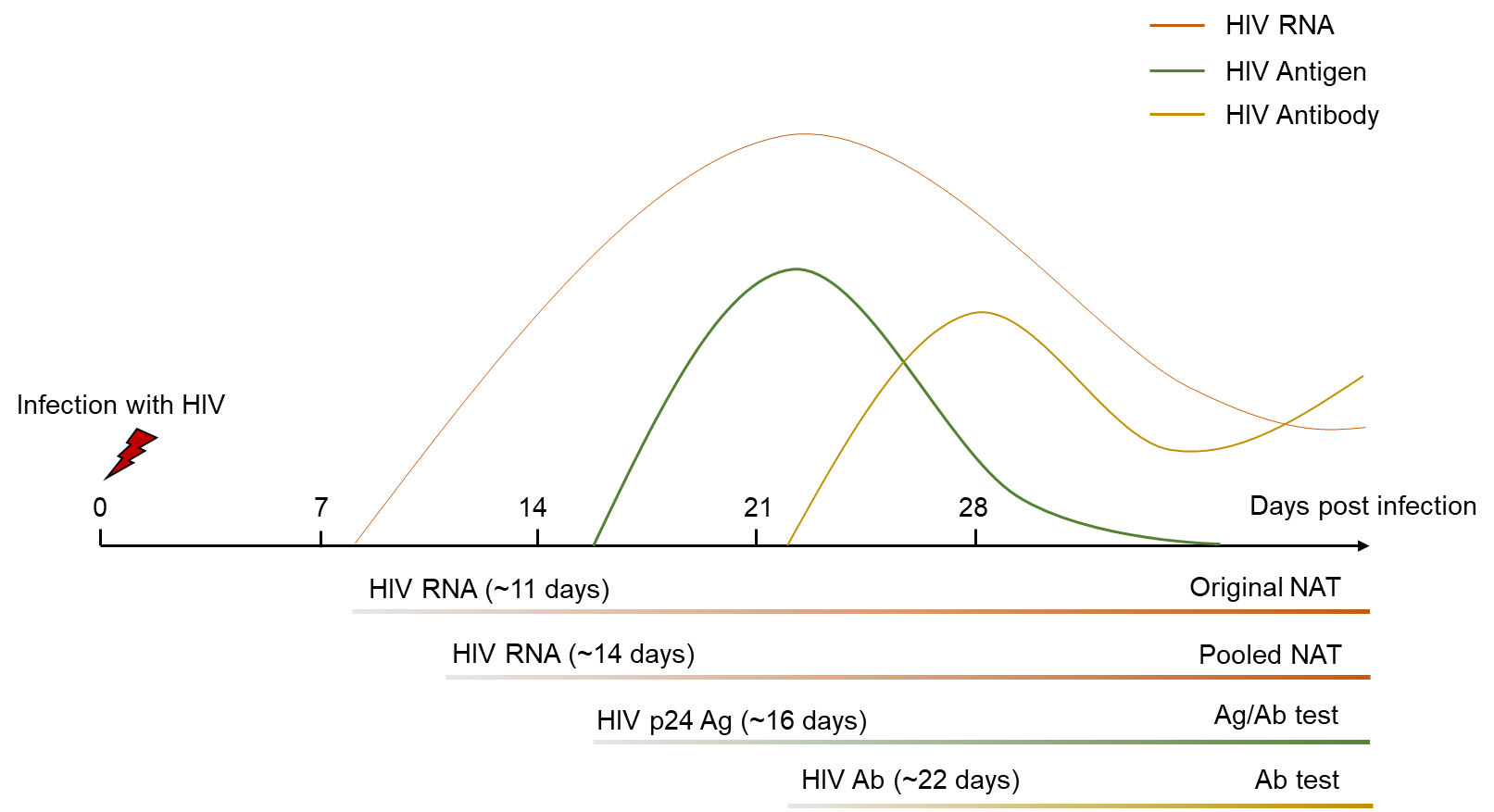


Supplementary Figure S1. Detection window of the HIV RNA, p24 antigen and antibody.

HIV, human immunodeficiency virus; Ab, antibodies; Ag, antigens; NAT, nucleic acid test.
