# Supplementary material for: Evaluation of the MAGLUMI HIV Ab/Ag combi test for the detection of HIV infection
Source: Virol J. 2024 Nov 13;21:290. doi: 10.1186/s12985-024-02565-x (PMC11562348; doi:10.1186/s12985-024-02565-x)
Supplement: Supplementary file 2 — Supplementary material 2. [file 12985_2024_2565_MOESM2_ESM.docx]

Supplementary Figure S2. Correlation of the results obtained on HIV-1 Ab (N=436), HIV-2 Ab (N=55) and HIV-1 Ag (N=50) positive samples between the MAGLUMI HIV Ab/Ag Combi and the Architect HIV Ag/Ab Combo.

HIV, human immunodeficiency virus; Ab, antibodies; Ag, antigens; AU, arbitrary unit; S/CO, signal/cut-off.
