# Supplementary material for: Evaluation of the MAGLUMI HIV Ab/Ag combi test for the detection of HIV infection
Source: Virol J. 2024 Nov 13;21:290. doi: 10.1186/s12985-024-02565-x (PMC11562348; doi:10.1186/s12985-024-02565-x)
Supplement: Supplementary file 3 — Supplementary material 3. [file 12985_2024_2565_MOESM3_ESM.docx]

| Sample ID | MAGLUMI HIV Ab/Ag Combi  (Cut-off = 1.0 AU/mL) | | |
| --- | --- | --- | --- |
|  | Initial | Repeat 1 | Repeat 2 |
| 1171469830 | 1.027 | 0.227 | 0.251 |
| 1171501260 | 1.957 | 1.906 | 1.738 |
| 1171512420 | 2.232 | 0.265 | 0.209 |
| 1171565700 | 3.430 | 0.144 | 0.178 |
| 1171579880 | 2.675 | 0.250 | 0.232 |
| 1171580030 | 1.162 | 0.280 | 0.266 |
| 1171602730 | 2.001 | 0.311 | 0.333 |
| 1171498990 | 1.403 | 0.400 | 0.320 |

Supplementary Table S1. Summary of results for eight presumed false positive blood donor samples.

HIV, human immunodeficiency virus; Ab, antibodies.
