# Supplementary material for: Evaluation of the MAGLUMI HIV Ab/Ag combi test for the detection of HIV infection
Source: Virol J. 2024 Nov 13;21:290. doi: 10.1186/s12985-024-02565-x (PMC11562348; doi:10.1186/s12985-024-02565-x)
Supplement: Supplementary file 4 — Supplementary material 4. [file 12985_2024_2565_MOESM4_ESM.docx]

| Panel ID | 1st positive day | | | |
| --- | --- | --- | --- | --- |
|  | MAGLUMI HIV Ab/Ag Combi | Architect HIV Ag/Ab Combo | Difference between two assays |  |
| PRB945 | 13 | 13 | 0 |  |
| PRB955 | 3 | 3 | 0 |  |
| PRB963 | 17 | 17 | 0 |  |
| PRB966 | 44 | 44 | 0 |  |
| PRB968 | 26 | 26 | 0 |  |
| PRB969 | 63 | 63 | 0 |  |
| PRB973 | 7 | 7 | 0 |  |
| HIV6244 | 28 | 28 | 0 |  |
| HIV6248 | 18 | 18 | 0 |  |
| HIV9011 | 38 | 38 | 0 |  |
| HIV9012 | 16 | 16 | 0 |  |
| HIV9013 | 23 | 25 | -2 |  |
| HIV9016 | 30 | 30 | 0 |  |
| HIV9018 | 28 | 28 | 0 |  |
| HIV9020 | 90 | 90 | 0 |  |
| HIV9021 | 47 | 47 | 0 |  |
| HIV9022 | 25 | 25 | 0 |  |
| HIV9023 | 78 | 78 | 0 |  |
| HIV9030 | 47 | 47 | 0 |  |
| HIV9031 | 138 | 146 | -8 |  |
| HIV9034 | 46 | 46 | 0 |  |
| HIV9076 | 66 | 66 | 0 |  |
| HIV9077 | 45 | 45 | 0 |  |
| HIV9079 | 40 | 40 | 0 |  |
| HIV9089 | 16 | 16 | 0 |  |
| HIV9096 | 3 | 3 | 0 |  |
| HIV12008 | 28 | 28 | 0 |  |
| SCP-HIV1-002 | 63 | 63 | 0 |  |
| SCP-HIV1-007 | 12 | 12 | 0 |  |
| 0600-0271 | 7 | 7 | 0 |  |
| Total delayed days on 30 panels | 1,105 | 1,115 | -10 |  |

Supplementary Table S2. Comparison of 1st positive day obtained with the MAGLUMI HIV Ab/Ag Combi and the Architect test.

HIV, human immunodeficiency virus; Ab, antibodies; Ag, antigens.
