# Supplementary material for: Evaluation of the MAGLUMI HIV Ab/Ag combi test for the detection of HIV infection
Source: Virol J. 2024 Nov 13;21:290. doi: 10.1186/s12985-024-02565-x (PMC11562348; doi:10.1186/s12985-024-02565-x)
Supplement: Supplementary file 5 — Supplementary material 5. [file 12985_2024_2565_MOESM5_ESM.docx]

| Sample ID  (panel ID-(number)) | MAGLUMI HIV Ab/Ag Combi | Architect HIV Ag/Ab Combo | HIV Ag^*^ | RNA^**^ | CONF^***^ |
| --- | --- | --- | --- | --- | --- |
|  | + | + | + | + | +(IND) |
| PRB945-(1-5) | 2 | 2 | 3 | 5 | 0 |
| PRB955-(1-4) | 3 | 3 | 3 | 4 | 0(4) |
| PRB963-(5-7) | 2 | 2 | 2 | 3 | 0 |
| PRB966-(6-10) | 3 | 3 | 3 | 5 | 0 |
| PRB968-(5-7) | 1 | 1 | 1 | 3 | 0 |
| PRB969-(4-7) | 1 | 1 | 1 | 4 | 0 |
| PRB973-(1-4) | 2 | 2 | 2 | 4 | 0 |
| HIV6244-(11-13) | 1 | 1 | 1 | 3 | 0(3) |
| HIV6248-(5-7) | 2 | 2 | 2 | 3 | 0 |
| HIV9011-(1-9) | 0 | 0 | 1 | 9 | 0(1) |
| HIV9012-(1, 3-7) | 2 | 2 | 3 | 6 | 0 |
| HIV9013-(5-7) | 2 | 1 | 2 | 3 | 0(3) |
| HIV9016-(8-10) | 2 | 2 | 2 | 3 | 0 |
| HIV9018-(7-10) | 2 | 2 | 2 | 4 | 0 |
| HIV9020-(18-22) | 3 | 3 | 4 | 5 | 0 |
| HIV9021-(13-17) | 4 | 4 | 4 | 5 | 0 |
| HIV9022-(6-8) | 1 | 1 | 2 | 3 | 0 |
| HIV9023-(19-22) | 3 | 3 | 3 | 4 | 0 |
| HIV9030-(12-16) | 3 | 3 | 3 | 5 | 0 |
| HIV9031-(5, 14-17) | 2 | 1 | 1 | 5 | 0 |
| HIV9034-(10-13) | 3 | 3 | 3 | 4 | 0 |
| HIV9076-(5, 6, 8, 9) | 2 | 2 | 2 | 4 | 0 |
| HIV9077-(11-14) | 3 | 3 | 3 | 4 | 0 |
| HIV9079-(8-11) | 3 | 3 | 3 | 4 | 0 |
| HIV9089-(3-5) | 2 | 2 | 2 | 3 | 0 |
| HIV9096-(1-5) | 4 | 4 | 4 | 5 | 0 |
| HIV12008-(7-10) | 2 | 2 | 2 | 4 | 0 |
| Total 114 samples | 60 | 58 | 64 | 114 | 0(11) |

Supplementary Table S3. Summary of results for the early seroconversion HIV samples.

^*^Result of the most sensitive p24 test mentioned on the seroconversion panel datasheet.

^**^Result of the most sensitive RNA test mentioned on the seroconversion panel datasheet.

^***^Result of the most sensitive Ab confirmation test mentioned on the seroconversion panel datasheet.

Column with header ‘+’ gives the number of tests with a positive result.

HIV, human immunodeficiency virus; Ab, antibodies; Ag, antigens; CONF, confirmatory assay; IND, indeterminate.

Abbott ARCHITECT

anti-HCV

MAGLUMI

anti-HCV Test (CLIA)
