# Supplementary material for: Evaluation of the MAGLUMI HIV Ab/Ag combi test for the detection of HIV infection
Source: Virol J. 2024 Nov 13;21:290. doi: 10.1186/s12985-024-02565-x (PMC11562348; doi:10.1186/s12985-024-02565-x)
Supplement: Supplementary file 6 — Supplementary material 6. [file 12985_2024_2565_MOESM6_ESM.docx]

| National reference | Measure | Acceptance criteria | MAGLUMI HIV Ab/Ag Combi |
| --- | --- | --- | --- |
| HIV-1 Ab | Negative coincidence rate | 13/13 | 13/13 |
|  | Positive coincidence rate | HIV-1 Group M: 14/14; | 14/14; |
|  |  | HIV-1 Group O: ≥1/3; | ≥1/3; |
|  |  | HIV-2: ≥2/3 | ≥2/3 |
|  | LoD reference materials (B/B’ subtype) | ≥3 react samples (≥3/5) | 5/5 |
|  | LoD reference materials (CRF_BC) | ≥3 react samples (≥3/5) | 4/5 |
|  | LoD reference materials (CRF_AE) | ≥3 react samples (≥3/5) | 3/5 |
|  | Precision reference materials | CV≤15% | CV=4.92% |
| HIV-1 p24 antigen | Negative coincidence rate | 20/20 | 20/20 |
|  | Positive coincidence rate | 10/10 | 10/10 |
|  | Linearity and LoD reference materials | LoD≤2.5  IU/mL | LoD=1.153 IU/mL |
|  | Precision reference materials | CV≤15% | CV=2.93% |

Supplementary Table S4. Analytical sensitivity in China National Reference for HIV-1 p24 antigen and HIV-1 antibody obtained with the MAGLUMI HIV Ab/Ag Combi.

HIV, human immunodeficiency virus; Ab, antibodies; LoD, Limit of Detection; CRF, circulating recombinant form.
