# Supplementary material for: Evaluation of the MAGLUMI HIV Ab/Ag combi test for the detection of HIV infection
Source: Virol J. 2024 Nov 13;21:290. doi: 10.1186/s12985-024-02565-x (PMC11562348; doi:10.1186/s12985-024-02565-x)
Supplement: Supplementary file 7 — Supplementary material 7. [file 12985_2024_2565_MOESM7_ESM.docx]

| National Reference | | | Sample | MAGLUMI HIV Ab/Ag Combi  AU/mL | |
| --- | --- | --- | --- | --- | --- |
| HIV-1 Ab LoD | B/B’ subtype | SB1 | | | 64.1 |
|  |  | SB2 | | | 33.2 |
|  |  | SB3 | | | 15 |
|  |  | SB4 | | | 5.47 |
|  |  | SB5 | | | 3.49 |
|  | CRF_BC | SBC1 | | | 17.6 |
|  |  | SBC2 | | | 6.81 |
|  |  | SBC3 | | | 4.67 |
|  |  | SBC4 | | | 1.59 |
|  |  | SBC5 | | | 0.652 |
|  | CRF_AE | SAE1 | | | 7.71 |
|  |  | SAE2 | | | 3.8 |
|  |  | SAE3 | | | 1.35 |
|  |  | SAE4 | | | 0.787 |
|  |  | SAE5 | | | 0.314 |
| HIV-1 p24 antigen linearity and LoD | | | L1 | | 14.6 |
|  |  |  | L2 | | 6.55 |
|  |  |  | L3 | | 3.2 |
|  |  |  | L4 | | 1.153 |
|  |  |  | L5 | | 0.543 |
|  |  |  | L6 | | 0.14 |
|  |  |  | L7 | | 0.184 |
|  |  |  | L8 | | 0.01 |
|  |  |  | L9 | | 0.01 |
|  |  |  | L10 | | 0.01 |

Supplementary Table S5. Analytical sensitivity in LoD reference materials of China National Reference for HIV-1 p24 antigen and HIV-1 antibody obtained with the MAGLUMI HIV Ab/Ag Combi.

HIV, human immunodeficiency virus; Ab, antibodies; Ag, antigens; AU, arbitrary unit; LoD, Limit of Detection.
